# Judicialización y acceso a tecnologías sanitarias: oportunidades y riesgos

**DOI:** 10.26633/RPSP.2017.137

**Published:** 2017-12-05

**Authors:** Jaume Vidal, y José Luis Di Fabio

**Affiliations:** 1 Health Action International (HAI) Health Action International (HAI) Health Action International (HAI), Ámsterdam, Países Bajos.; 2 Organización Panamericana de la Salud Organización Panamericana de la Salud Washington D.C. Estados Unidos de América Organización Panamericana de la Salud, Washington D.C., Estados Unidos de América.

**Keywords:** acceso a la atención de salud, poder judicial, legislación, jurisprudencia, Pharmaceutical preparations, health services accessibility, judiciary, legislation, jurisprudence, Preparações farmacéuticas, acesso aos serviços de saúde, poder judiciário, legislação, jurisprudência

## Abstract

*La utilización de la vía judicial para asegurar y garantizar el acceso a insumos médicos constituye una de las estrategias más efectivas para asegurar el cumplimiento (o remediar la violación) del derecho a la salud, a menudo en relación con otros derechos humanos fundamentales como el derecho a la vida o a la integridad física*.

*De manera más frecuente y en múltiples contextos nacionales, jueces y cortes respaldan las demandas interpuestas por particulares que necesitan acceso inmediato a tecnologías sanitarias. En muchas ocasiones, las senten-cias que obligan al Estado y a sus instituciones a garantizar el suministro de un producto determinado no toman en consideración las razones aducidas por el Estado para no suministrarlo que pueden variar desde cálculos de coste-efectividad a procesos de evaluación, planificación y ejecución de políticas públicas*.

*La utilización y abuso de procedimientos judiciales por terceras partes interesadas amenaza la legitimidad de un instrumento que ha contribuido, sin lugar a dudas, a fortalecer la participación de la ciudadanía en la defensa de sus derechos, incluido el derecho a la salud*.

*El presente documento pretende contextualizar la evolución del fenómeno en relación con aquellos instrumentos, mecanismos y procedimientos que suelen utilizar las autoridades sanitarias para racionalizar el acceso a tecnologías sanitarias. Además, se sugieren pasos a seguir tanto en el ámbito nacional como regional*.

## Abstract (en)

Use of litigation to ensure and guarantee access to medical inputs constitutes one of the most effective strategies to ensure observance (or remedy the violation) of the right to health, often in relation to other fundamental human rights such as the right to life or to bodily integrity.

More frequently, and in multiple national contexts, judges and courts support lawsuits filed by individuals who need immediate access to health technologies. On many occasions, the rulings that force the State and its institutions to guarantee the supply of a given product do not take into account the reasons the State gives for not providing it, which can range from cost-effectiveness calculations and evaluation, to public policy planning and implementation.

Use and abuse of legal proceedings by interested third parties threaten the legitimacy of an instrument that has contributed, without a doubt, to strengthening public engagement in the defense of people’s rights, including the right to health.

This document is an attempt to provide a context for the evolution of this phenomenon with regard to the instruments, mechanisms, and procedures commonly used by health authorities to efficiently organize access to health technologies. In addition, steps to follow are suggested for both national and regional settings.

## RESUMO

A utilização da via judicial para assegurar e garantir o acesso a insumos médicos é uma das estratégias mais efetivas para o cumprimento (ou remediar o descumprimento) do direito à saúde, frequentemente relacionado a outros direitos humanos fundamentais como o direito à vida ou direito à integridade física.

Com frequência e em diversos contextos nacionais, juízes e tribunais dão respaldo aos recursos apresentados por indivíduos particulares que precisam ter acesso imediato a tecnologias em saúde. Em várias ocasiões, as sentenças que obrigam o Estado e suas instituições a garantir a provisão de determinado produto não levam em consideração as razões alegadas para a não provisão, de cálculos de custo-efetividade a processos de avaliação, planejamento e execução de políticas públicas.

O uso e o abuso de recursos judiciais por terceiros interessados ameaçam a legitimidade de um instrumento que vem indubitavelmente contribuindo para fortalecer a participação dos cidadãos na defesa dos próprios direitos, inclusive o direito à saúde.

O presente documento tem o intuito de contextualizar a evolução do fenômeno da judicialização quanto aos instrumentos, mecanismos e procedimentos normalmente usados pelas autoridades sanitárias para racionalizar o acesso a tecnologias em saúde. É feita recomendação sobre os passos a serem seguidos ao nível nacional e regional.

En un fenómeno cuyo orígenes se encuentran en la lucha por conseguir acceso a medicamentos antirretrovirales a principios de los 90, fortalecido por la demanda de otros medicamentos (oncológicos y contra la hepatitis C), cortes y jueces se han consolidado como factores sustantivos en la garantía del derecho humano a la salud (o en el remedio de su violación) cuando su realización depende del acceso a tecnologías sanitarias. Sus decisiones, sin embargo, pueden menoscabar la capacidad y competencia de las autoridades sanitarias.

Así, y con especial intensidad en algunos países de la Región de las Américas, las autoridades judiciales respaldan las demandas interpuestas por particulares que necesitan acceso inmediato a tecnologías sanitarias (1, 2). En ocasiones, las sentencias que obligan al Estado y a sus instituciones a garantizar el suministro de un producto determinado no toman en suficiente consideración las razones aducidas por el Estado en su momento para no suministrarlo (o no subsidiar su compra). Estas razones van desde estimaciones de coste-efectividad y procesos de evaluación de tecnologías sanitarias (ETS) a exclusión de la lista oficial de medicamentos (LOM) y decisiones de asignación de recursos asociadas a la pla-nificación y ejecución de políticas públicas.

## Judicialización: concepto, contexto legal y desarrollo

Se entiende por judicialización del acceso a tecnologías sanitarias a la utilización por parte de la ciudadanía de instrumentos legales de participación para interpelar al estamento judicial en caso de violación percibida de su derecho a la salud, debido a la falta de acceso a tratamiento médico o farmacológico en el sistema público de salud o “aquellas situaciones en que el Poder Judicial toma parte de las decisiones que en materia de salud normalmente competen a otros poderes o instancias del Estado, específicamente a instituciones del sector salud” (3).

El recurso a las cortes por parte de sujetos individuales en defensa de sus derechos humanos en general y en concreto del derecho humano a la salud solo es po-sible por la codificación y reconocimiento de estos derechos en el ámbito internacional y su introducción mediante ratificación y posterior reforma constitucional en el ordenamiento jurídico interno.

Existe un amplio conjunto de tratados y convenciones internacionales (y regionales) que, ratificados por la gran mayoría de Estados, establecen la naturaleza y características del derecho humano a la salud (4). Su definición más ampliamente aceptada se encuentra en el Pacto Internacional para los Derechos Económicos, Sociales y Culturales (PIDESC), que reconoce en su artículo 12 “el derecho de toda persona al disfrute del más alto nivel posible de salud física y mental”; una interpretación autorizada de sus características así como de su articulación con el acceso a tecnologías sanitarias se encuentra en la Observación General Número 14 del Comité Internacional del PIDESC (5–7).

## Judicialización y derecho a la salud

El fenómeno de la judicialización no se manifiesta de manera uniforme en todos los países, con una mayoría de casos registrada en la región de las Américas. Este desequilibrio obedece a varios elementos, entre los que destacan: las características del régimen jurídico imperante, especialmente la extensión de la autoridad judicial (8); la posibilidad legal, reconocida en la Constitución, para la ciudadanía de emplazar judicialmente, vía recurso individual de amparo o tutela, ante la Suprema Corte o cámaras habilitadas, al Estado en caso de violación a sus derechos humanos o constitucionales (9, 10) y, por último, el hecho de que un gran número de constituciones nacionales en las Américas reconocen el derecho a la salud, en ocasiones en relación con el derecho a la vida y la integridad física y, en algunos casos, con explícitas referencias al acceso a tecnologías sanitarias (11). No obstante, debe notarse que, en gran parte de los recursos presentados, el principal argumento no es una violación al derecho a la salud, sino una amenaza al derecho a la vida y la integridad física por vulneración del primero de la persona o grupo de personas que inicia el proceso, con la sentencia aplicada solo a estas.

Tales factores explicarían por qué la prevalencia de la judicialización es notablemente más alta en algunos países cuyas jurisdicciones han acumulado, en los últimos 15 años, una prolífica jurisprudencia de sentencias que obligan al Estado, en nombre de la protección del derecho a la salud y otros derechos humanos, a garantizar el acceso efectivo a diversos tratamientos farmacológicos (12). Se destacan los casos de Brasil, donde el monto de recursos estatales asignados al cumplimiento de sentencias judiciales relacionadas con recursos de amparo se incrementó de manera exponencial entre 2005 y 2010 (con un gasto derivado en medicamentos aquel año de 550 millones de dólares estadounidenses a nivel federal); Colombia, donde el ministerio de salud estimaba en 300 millones de dólares estadounidenses el coste directo ligado a juicios por judicialización del acceso a medicamentos; o Costa Rica, donde entre 1999 y 2008 se registraron 2 524 recursos de amparo para reclamar el acceso a medicamentos mientras un aumento sustantivo respecto al período 1989-1998, durante el cual se habían presentado 178 (13-15). Este aumento de casos y sentencias ha consolidado la intervención judicial en el ámbito médico-sanitario, con consecuencias institucionales y presupuestarias para los sistemas públicos de salud.

Los procesos de judicialización, por las características de sus procedimientos legales, se han basado en gran medida en una visión individual donde el no respeto al derecho a la salud comportaría una violación del derecho a la vida. Esta interpretación del derecho a la salud omite una visión más amplia de la salud como bien colectivo, cuya mejor protección son sistemas públicos efectivos, eficaces y necesariamente sostenibles para poder dar el mejor servicio al conjunto amplio de la población. En última instancia, los procesos de judicialización pueden suponer una amenaza a la equidad de los sistemas de salud, ya que afectan la provisión de productos y servicios para toda la ciudadanía, más allá de los casos individuales examinados por cortes y jueces (16, 17).

## Derecho a la salud: obligaciones y límites de las acciones del Estado

El respeto, protección y promoción del derecho a la salud son una responsabilidad exclusiva del Estado, del conjunto de instituciones y agentes. El papel principal de la vía judicial es proteger a la víctima y ofrecer remedio ante un eventual quebranto de disposiciones legales, incluidos tratados internacionales recogidos en la Constitución. El rol del Estado va más allá y, como se señaló en su momento en la Observación General No. 14, implica la adopción de políticas y acciones que promuevan el derecho a la salud y que contrarresten eventuales impactos negativos de la acción de terceros sobre él (6).

En términos de salud pública, esto abarca todo el conjunto de actividades y acciones requeridas tanto para la prevención de posibles enfermedades como la promoción de las capacidades del Estado para dar respuesta a las mismas. Se incluyen, de forma no exhaustiva, la inversión en equipos e instalaciones, la capacitación del personal sanitario, la diseminación de información relevante para pacientes y cuidadores, la regulación y monitoreo de insumos y equipos y todo esfuerzo para obtener el máximo y mejor resultado de los recursos públicos destinados a la compra o subsidio de tecnologías sanitarias.

Esto significa que forma parte del compromiso del Estado para con el respeto, promoción y protección del derecho a la salud el garantizar una autoridad nacional reguladora efectiva, el establecimiento de protocolos sistemáticos de ETS (18), políticas de promoción de uso racional del medicamento y de receta por denominación común internacional (DCI), así como el impulso a la sustitución por versiones genéricas o biosimilares siempre que sea posible (19) entre las medidas consideradas como más eficaces para contener costos y garantizar la viabilidad financiera de los sistemas públicos de salud.

## Propuestas y objetivos

Se hace necesaria una reinterpretación de la judicialización del acceso a medicamentos desde una perspectiva sanitaria, de manera que eventuales respuestas a demandas individuales no menoscaben principios fundamentales de equidad o erosionen la efectividad de mecanismos como la ETS o de instrumentos como la LOM antes mencionados (18, 19). Además, la utilización y abuso de procedimientos judiciales por terceras partes interesadas amenaza la legitimidad de un instrumento que ha contribuido, sin lugar a dudas, a fortalecer la participación de la ciudadanía en la defensa de sus derechos, incluido el derecho a la salud. Un esfuerzo, en suma, para integrar los procesos de judicialización en el espectro más amplio de interacciones entre el Estado y la ciudadanía.

No se trata, en ningún caso, de limitar derechos, sino de contextualizarlos en el marco médico-sanitario, con valoración de aquellos elementos ya existentes como la selección justificada en términos de coste-efectividad de medicamentos financiados por el Estado, énfasis en la importancia de recetar por DCI o el uso intensivo de las flexibilidades previstas en el Acuerdo sobre Derechos de Propiedad Intelectual aplicados al Comercio (ADPIC) en la concesión de patentes o aprobación regulatoria (20); en suma, un conjunto de medidas adoptadas por diversos órganos del Estado para asegurar no solo el derecho a la salud de cada uno de sus ciudadanos, sino la protección y promoción de la salud pública de toda la sociedad (21).

Resulta esencial que las autoridades sanitarias asuman los fallos judiciales dictados en su contra para mejorar su actuación futura, sobre todo en aquellas instancias donde la violación denunciada se debe a situaciones de desabastecimiento atribuibles a fallos de suministro o descoordinación entre centros. De igual manera, corresponde al sector salud explicar, de forma pública y accesible, la metodología, criterios y otros elementos que guían aquellas decisiones que afectan las condiciones de acceso a tecnologías sanitarias. Cabe recordar que el derecho a la información también forma parte de la realización del derecho a la salud (22).

Al mismo tiempo, hay que garantizar que los dictámenes técnicos sobre los que se basan las decisiones de las autoridades sanitarias para autorizar, financiar o racionar medicamentos u otras tecnologías sanitarias no puedan ser obviados de manera unilateral por la Corte y los jueces; siendo necesaria la protección de los criterios técnicos de decisión de los profesionales sanitarios responsables deben tener primacía sobre las opiniones de otros actores como, por ejemplo, el médico prescriptor. Este amparo a las decisiones técnicas de las autoridades sanitarias debe ser explicitado mediante la codificación en texto legal por parte del Poder Legislativo y refrendado por los órganos de interpretación del Poder Judicial.

Se anima a la posibilidad de explorar mecanismos de coordinación interinstitucional entre miembros del Poder Judicial (jueces y fiscales) y personal del sector salud (incluidos el ministerio de salud, los entes reguladores y las instituciones técnicas adscritas), en especial con responsabilidad en la selección y actualización de la LOM, examen y aprobación del registro sanitario, planificación y evaluación de políticas farmacéuticas, y desarrollo de evaluaciones de tecnologías sanitarias. El objetivo de estos ejercicios sería, en esencia, la discusión y elaboración de protocolos de actuación que permita habilitar espacios de conciliación y consulta paralelos a la resolución judicial de procesos de judicialización.

Por último, se recomienda igualmente que las autoridades sanitarias, con la colaboración de la academia y la sociedad civil organizada, abran un espacio de diálogo sobre las oportunidades que la judicialización puede representar para la mejora del desempeño del Estado en la defensa del derecho a la salud y, también, sobre la amenaza a la sostenibilidad financiera del sistema público de salud, y por ende a la equidad del mismo, su utilización sistemática, identificación de debilidades y fortalezas. Esto permitirá sugerir posibles cambios legales, administrativos e institucionales para dar una mejor respuesta a las necesidades de la ciudadanía en base al conjunto de mejores prácticas identificadas en contextos de recursos limitados (22). El producto resultante, una vez compiladas todas las contribuciones, podría desarrollarse como herramienta para la capacitación de funcionarios judiciales, sanitarios y de otras dependencias del Estado con responsabilidad en los diferentes aspectos del acceso a tecnologías sanitarias y como insumo para legisladores.

Correspondería a las agencias técnicas especializadas brindar el apoyo, el acompañamiento y la guía que las autoridades nacionales pudieran requerir tanto en la compilación y articulación de información como en la implementación de eventuales reformas o cambios jurídico-normativos.

### Declaración.

 Las opiniones expresadas en este manuscrito son responsabilidad del autor y no reflejan necesariamente los criterios ni la política de la RPSP/PAJPH y/o de la OPS
